# Supplementary material for: Silencing of STE20-type kinase STK25 in human aortic endothelial and smooth muscle cells is atheroprotective
Source: Commun Biol. 2022 Apr 19;5:379. doi: 10.1038/s42003-022-03309-9 (PMC9018782; doi:10.1038/s42003-022-03309-9)
Supplement: Supplementary file 2 — Supplementary Information [file 42003_2022_3309_MOESM2_ESM.pdf]

Supplementary information

Silencing of STE20-Type Kinase STK25 in Human Aortic Endothelial and Smooth Muscle Cells is Atheroprotective

Cansby et al.

Supplementary Figure 1

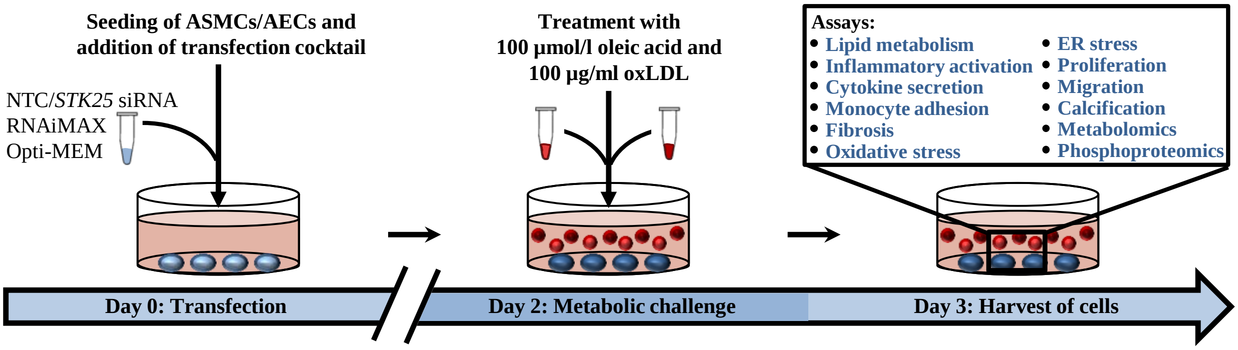

Supplementary Fig. 1. Schematic illustration of the study design.

Supplementary Figure 2

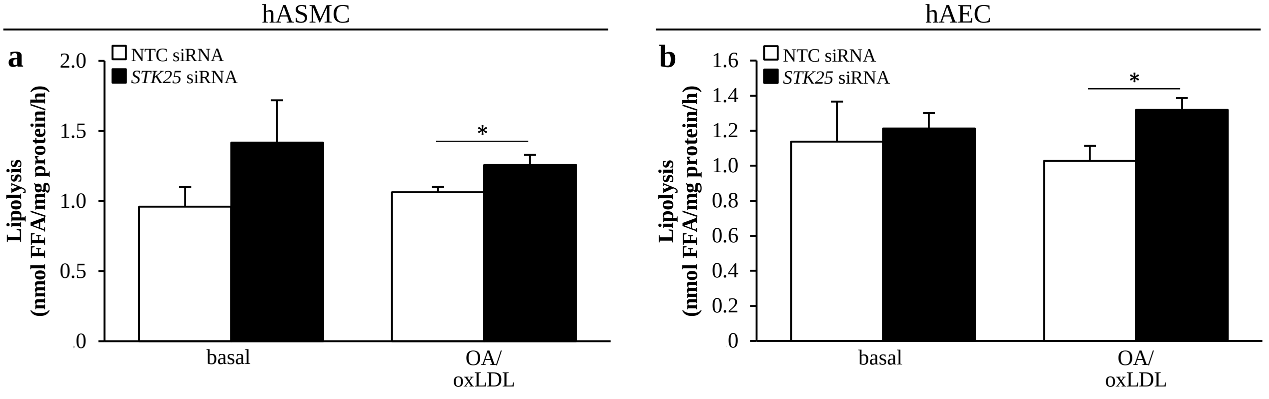

Supplementary Fig. 2. Silencing of STK25 increases lipolysis in human aortic endothelial and smooth muscle cells. hASMCs and hAECs were transfected with *STK25* siRNA or NTC siRNA. TAG hydrolase activity measured in hASMCs (a) and hAECs (b) using [^{3}H]triolein as the substrate. Data are mean ± SEM from 6 wells per group. FFA, free fatty acid; OA, oleic acid. *P<0.05 for *STK25* siRNA *vs*. NTC siRNA

Supplementary Figure 3

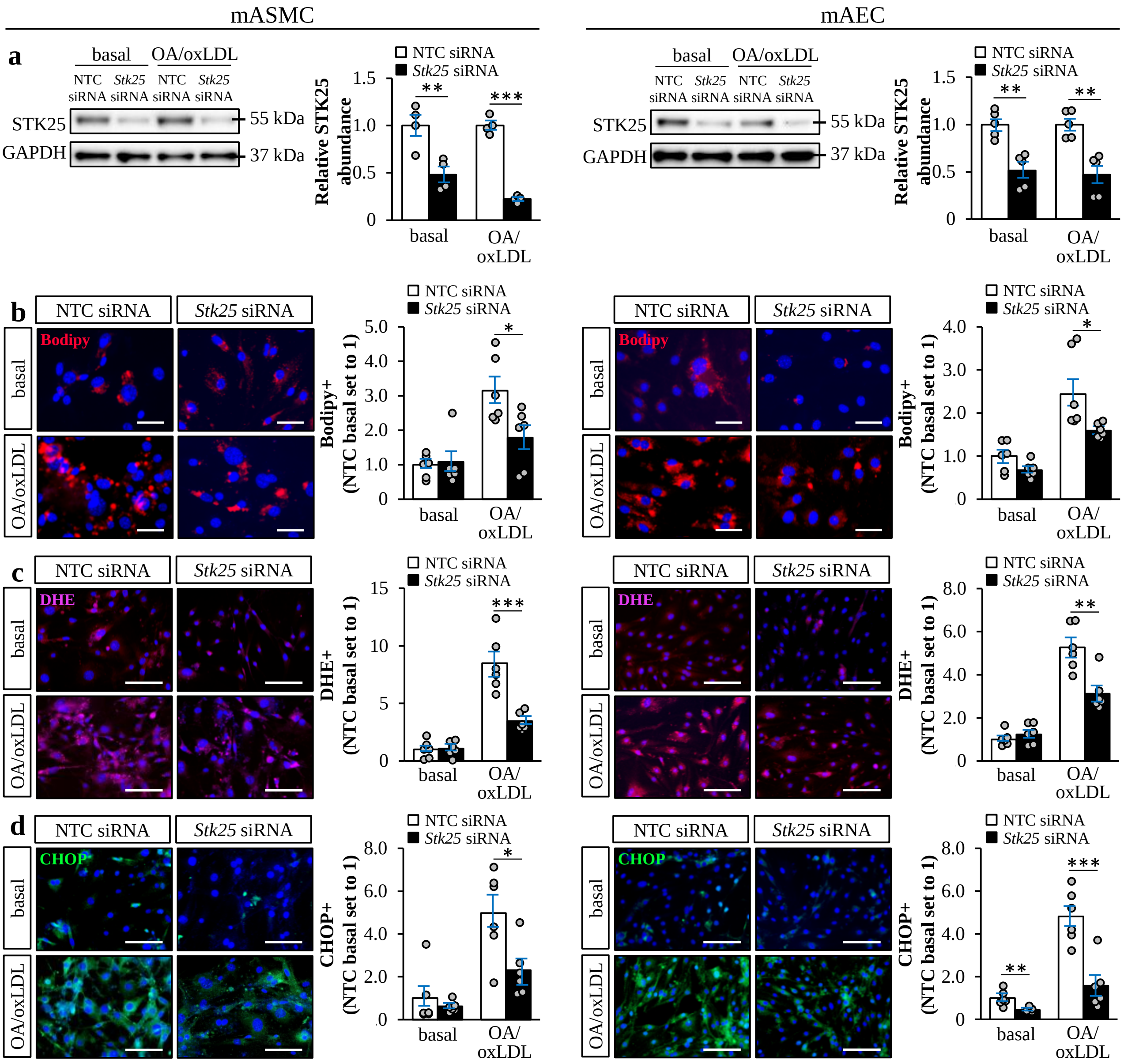

Supplementary Fig. 3. Silencing of STK25 suppresses lipid accumulation and oxidative and ER stress in mouse aortic endothelial and smooth muscle cells. mASMCs and mAECs were transfected with *Stk25* siRNA or NTC siRNA. (a) STK25 protein abundance. Protein levels were analyzed by densitometry; representative Western blots are shown with GAPDH used as a loading control. (b-d) Representative images of cells stained with Bodipy (red) or DHE (pink) or processed for immunofluorescence with anti-CHOP (green) antibody; nuclei stained with DAPI (blue). Quantification of the staining. In (b) and (c-d), the scale bars represent 25 and 100 µm, respectively. Data are mean ± SEM from 4-6 wells per group. OA, oleic acid. **P*<0.05, ***P*<0.01, ****P*<0.001 for *Stk25* siRNA *vs*. NTC siRNA

Supplementary Figure 4

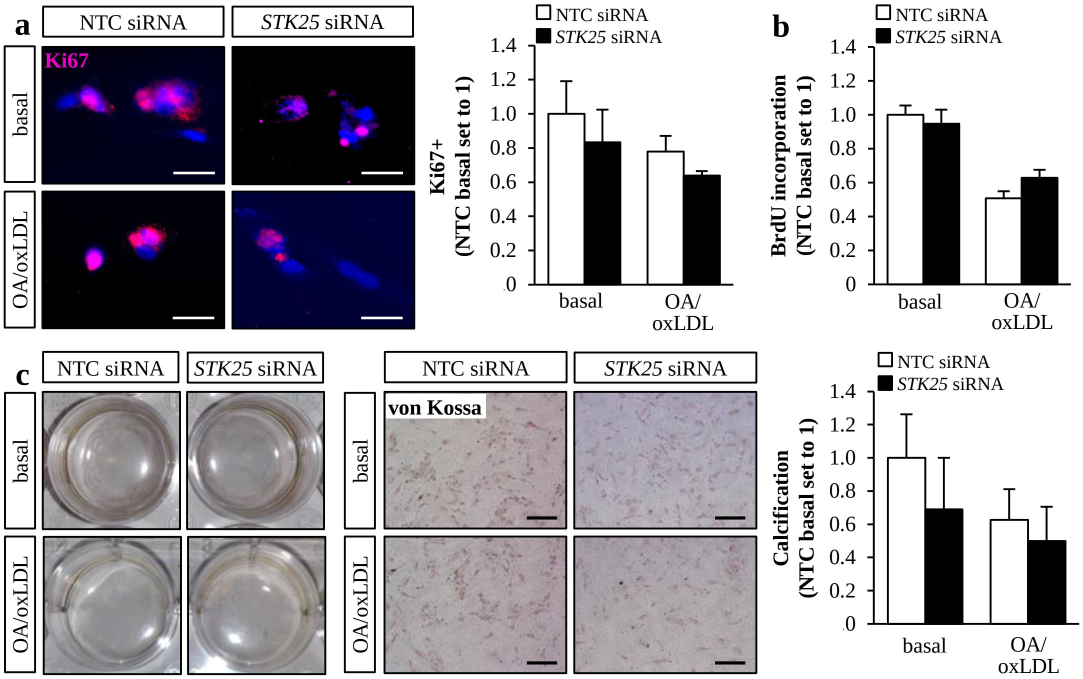

Supplementary Fig. 4. STK25 does not control proliferation or calcification in human aortic endothelial cells. hAECs were transfected with *STK25* siRNA or NTC siRNA. (a) Representative images of cells processed for immunofluorescence with anti-Ki67 (pink) antibody; nuclei stained with DAPI (blue). Quantification of the staining. (b) Proliferation rate assessed by measuring the DNA synthesis using a Biotrack cell proliferation ELISA. (c) Representative images of calcium deposition. Quantification of the staining. In (a) and (c), the scale bars represent 25 and 50 µm, respectively. Data are mean ± SEM from 6-8 wells per group. OA, oleic acid

Supplementary Figure 5

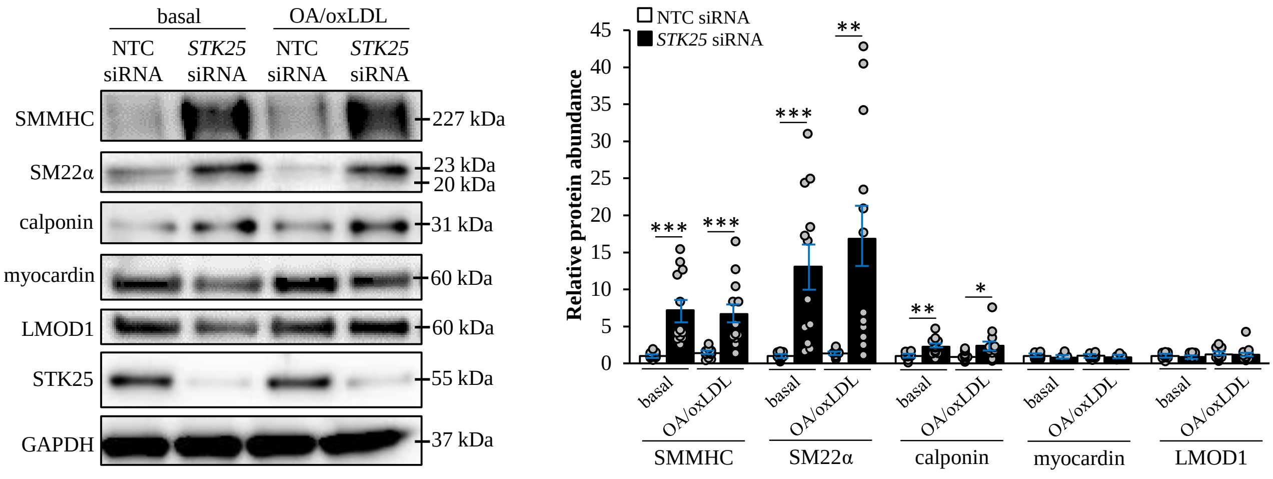

Supplementary Fig. 5. Silencing of STK25 increases the abundance of contractile markers in human aortic smooth muscle cells. hASMCs were transfected with *STK25* siRNA or NTC siRNA. Cell lysates were analyzed by Western blot using antibodies specific for SMMHC, SM22α, calponin, myocardin, LMOD1, and STK25. Protein levels were analyzed by densitometry; representative Western blots are shown with GAPDH used as a loading control. Data are mean ± SEM from 11-12 wells per group. OA, oleic acid. *P<0.05, **P<0.01, ***P<0.001 for *STK25* siRNA *vs*. NTC siRNA

Supplementary Figure 6

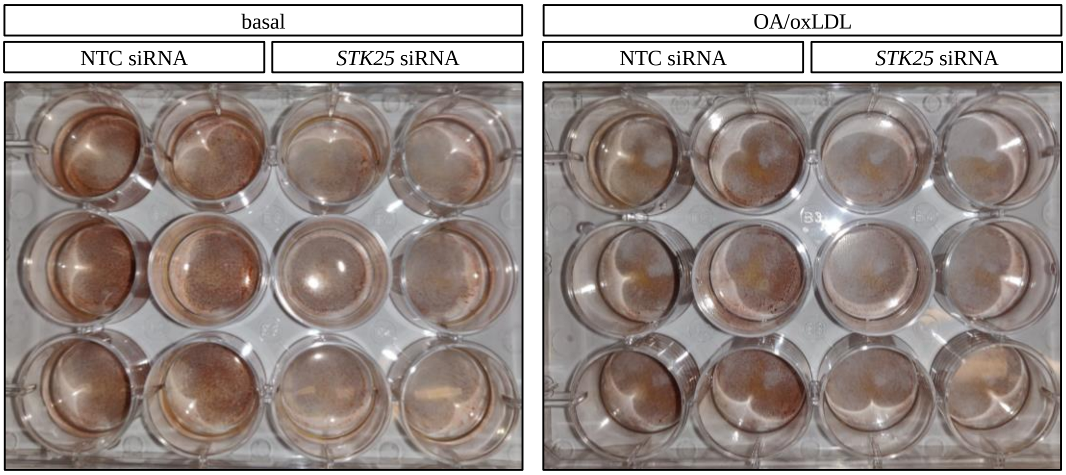

Supplementary Fig. 6. Silencing of STK25 decreases calcification in human aortic smooth muscle cells. hASMCs were transfected with *STK25* siRNA or NTC siRNA. Representative images of cells incubated with silver nitrate and exposed to light. OA, oleic acid

Supplementary Figure 7

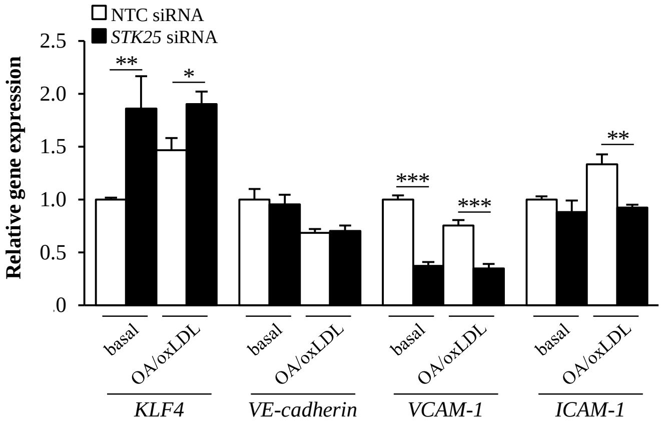

Supplementary Fig. 7. STK25 protects against dysfunctional changes in human aortic endothelial cells. hAECs were transfected with *STK25* siRNA or NTC siRNA. Gene expressions of selected markers of endothelial cell dysfunction. Data are mean ± SEM from 6 wells per group. OA, oleic acid. **P*<0.05, ***P*<0.01, ****P*<0.001 for *STK25* siRNA *vs*. NTC siRNA

Supplementary Figure 8

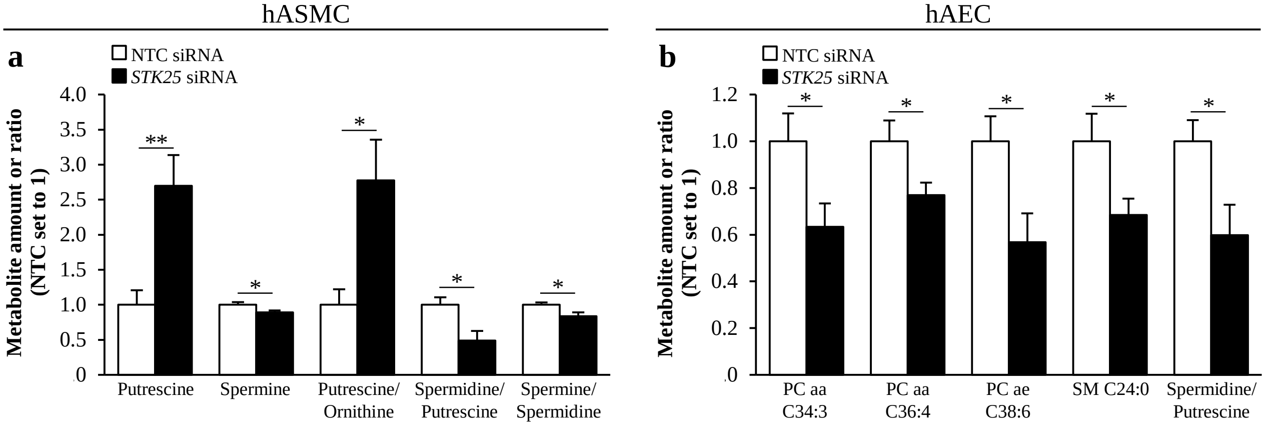

Supplementary Fig. 8. Silencing of STK25 alters the concentration of polyamines in human aortic endothelial and smooth muscle cells. hASMCs and hAECs were transfected with *STK25* siRNA or NTC siRNA. Cellular metabolite levels and ratios were measured in hASMCs (a) and hAECs (b) using the AbsoluteIDQ p180 Kit. Significant differences are shown. Data are mean ± SEM from 7-8 wells per group. **P*<0.05, ***P*<0.01 for *STK25* siRNA *vs*. NTC siRNA

Supplementary Figure 9

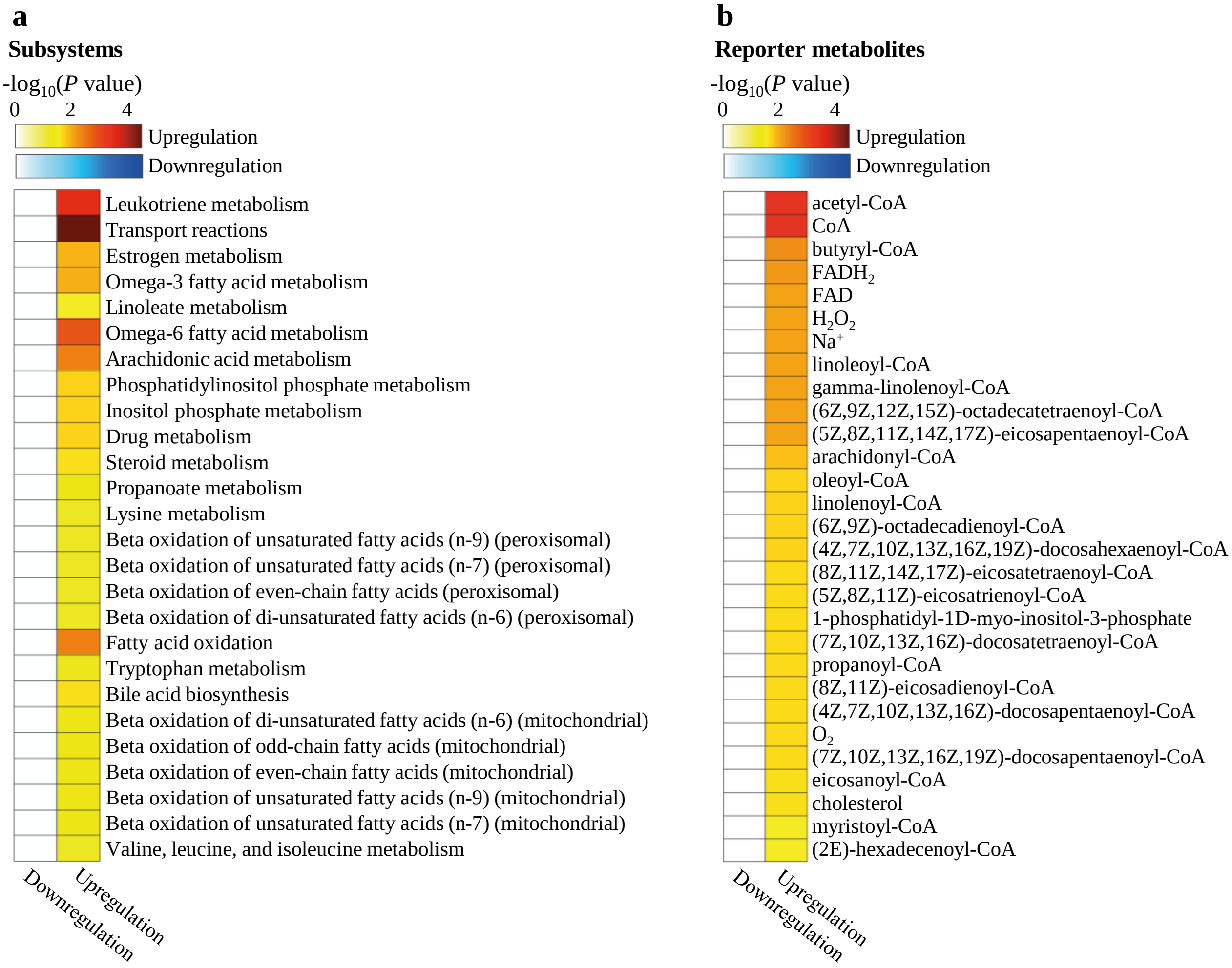

Supplementary Fig. 9. Enrichment analysis of subsystems and reporter metabolite sets was performed by integrative analysis of global phosphoproteomic data obtained from STK25-deficient hASMCs with the genome-scale metabolic model Human1.[61] Up- or downregulation (*P*<0.05) in subsystems (a) and reporter metabolite sets (b) in response to knockdown of STK25 are shown.

Supplementary Figure 10

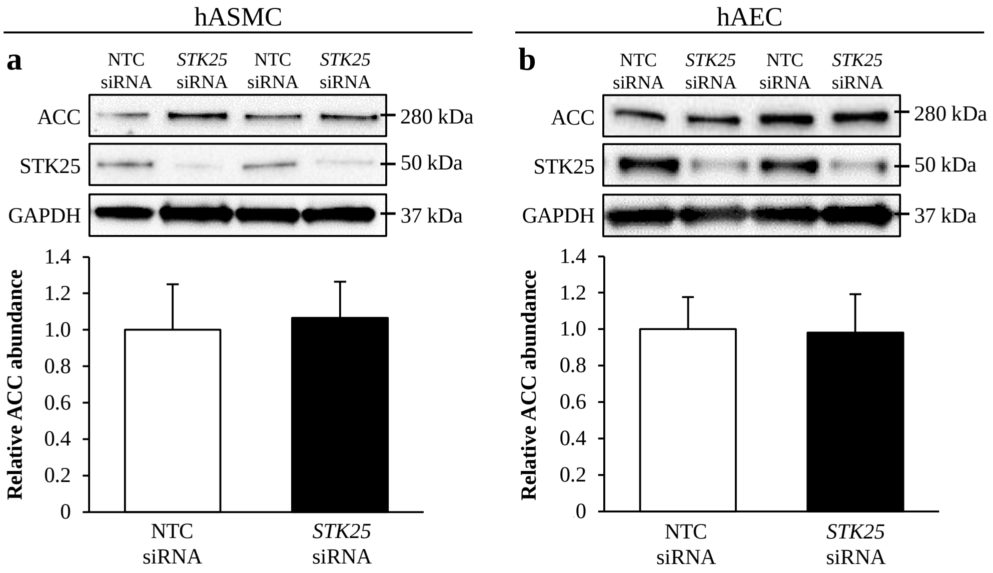

Supplementary Fig. 10. Silencing of STK25 does not affect the levels of ACC in human aortic endothelial and smooth muscle cells. hASMCs and hAECs were transfected with *STK25* siRNA or NTC siRNA. Cell lysates from hASMCs (a) and hAECs (b) were analyzed by Western blot using antibodies specific for ACC and STK25. Protein levels were analyzed by densitometry; representative Western blots are shown with GAPDH used as a loading control. Data are mean ± SEM from 6-9 wells per group.

Supplementary Figure 11

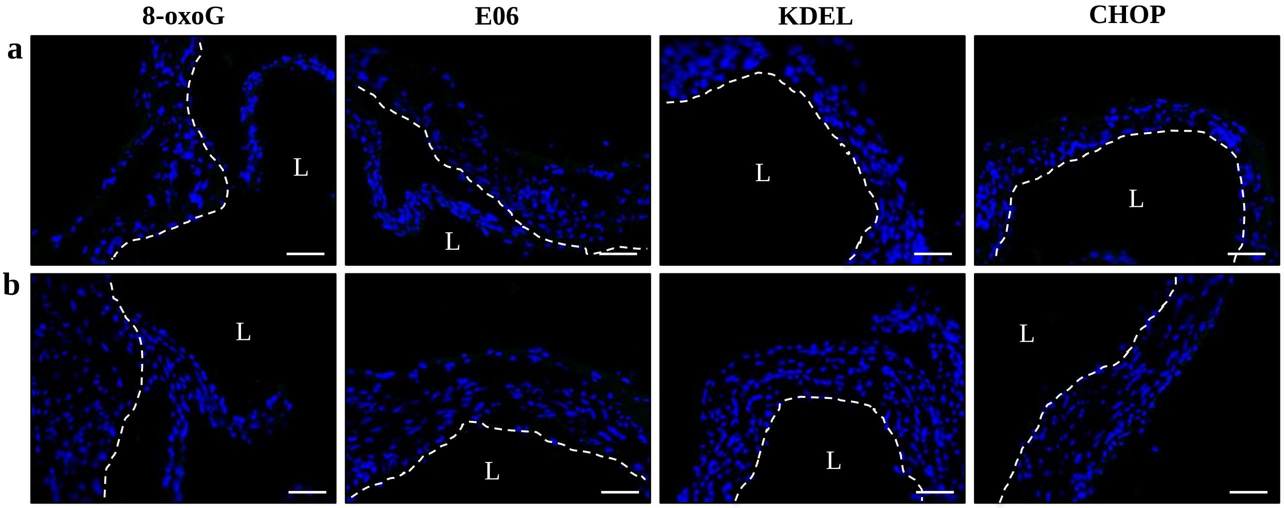

Supplementary Fig. 11. Representative negative control images for immunofluorescence analysis in sections of aortic sinus from atherosclerotic mice by excluding primary antibody (a) or by substituting a primary antibody by an equivalent concentration of the corresponding Ig isotype (b); nuclei stained with DAPI (blue). Dashed line and L indicate lumen. The scale bars represent 50 µm. L, lumen

Supplementary Figure 12

Figure 1b

1. NTC siRNA basal
2. *STK25* siRNA basal
3. NTC siRNA treated
4. *STK25* siRNA treated
5. NTC siRNA basal
6. *STK25* siRNA basal
7. NTC siRNA treated
8. *STK25* siRNA treated
9. NTC siRNA basal
10. *STK25* siRNA basal
11. NTC siRNA treated
12. *STK25* siRNA treated

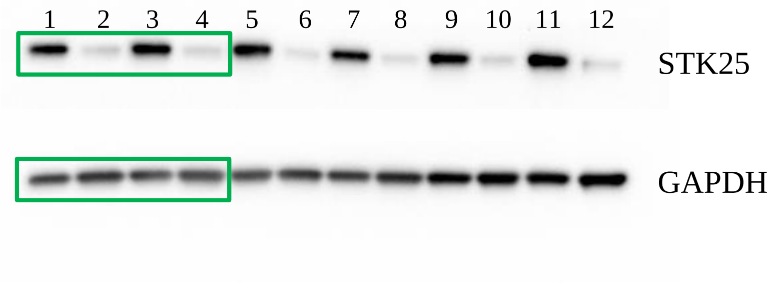

1. NTC siRNA basal
2. *STK25* siRNA basal
3. NTC siRNA treated
4. *STK25* siRNA treated

5-12. Experiment not related to this panel

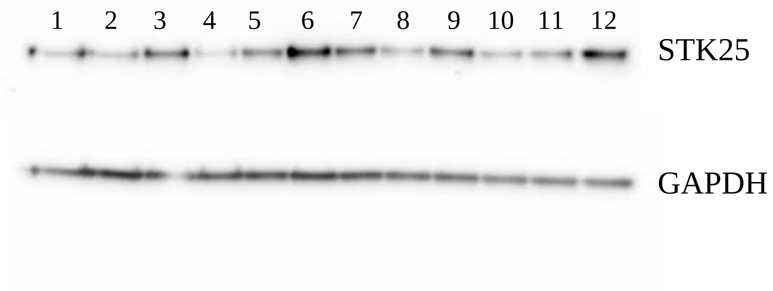

Figure 1i

1. NTC siRNA basal
2. *STK25* siRNA basal
3. NTC siRNA treated
4. *STK25* siRNA treated
5. NTC siRNA basal
6. *STK25* siRNA basal
7. NTC siRNA treated
8. *STK25* siRNA treated
9. NTC siRNA basal
10. *STK25* siRNA basal
11. NTC siRNA treated
12. *STK25* siRNA treated

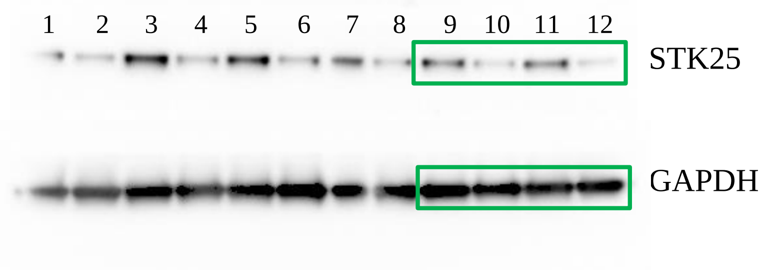

1-2. Experiment not related to this panel

7. NTC siRNA basal
8. *STK25* siRNA basal
9. NTC siRNA treated
10. *STK25* siRNA treated

11-12. Experiment not related to this panel

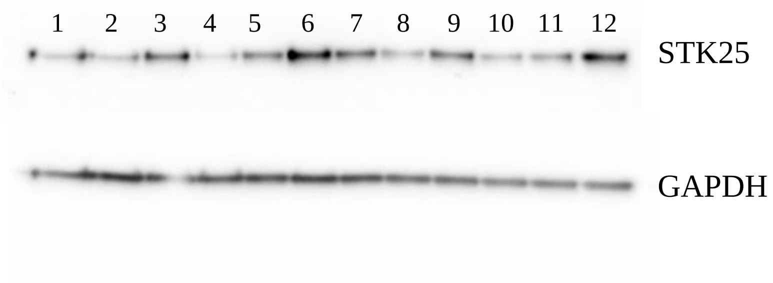

Green boxes indicates part shown in each respective figure

Figure 2b

1. NTC siRNA basal
2. *STK25* siRNA basal
3. NTC siRNA treated
4. *STK25* siRNA treated
5. NTC siRNA basal
6. *STK25* siRNA basal
7. NTC siRNA treated
8. *STK25* siRNA treated
9. NTC siRNA basal
10. *STK25* siRNA basal
11. NTC siRNA treated
12. *STK25* siRNA treated

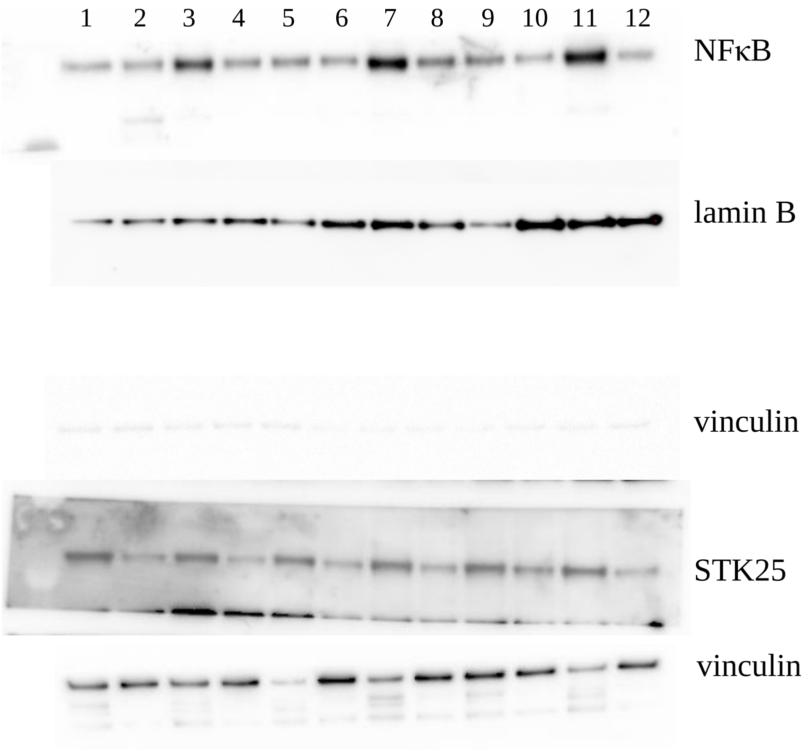

1. NTC siRNA basal
2. *STK25* siRNA basal
3. NTC siRNA treated
4. *STK25* siRNA treated
5. NTC siRNA basal
6. *STK25* siRNA basal
7. NTC siRNA treated
8. *STK25* siRNA treated
9. NTC siRNA basal
10. *STK25* siRNA basal
11. NTC siRNA treated
12. *STK25* siRNA treated

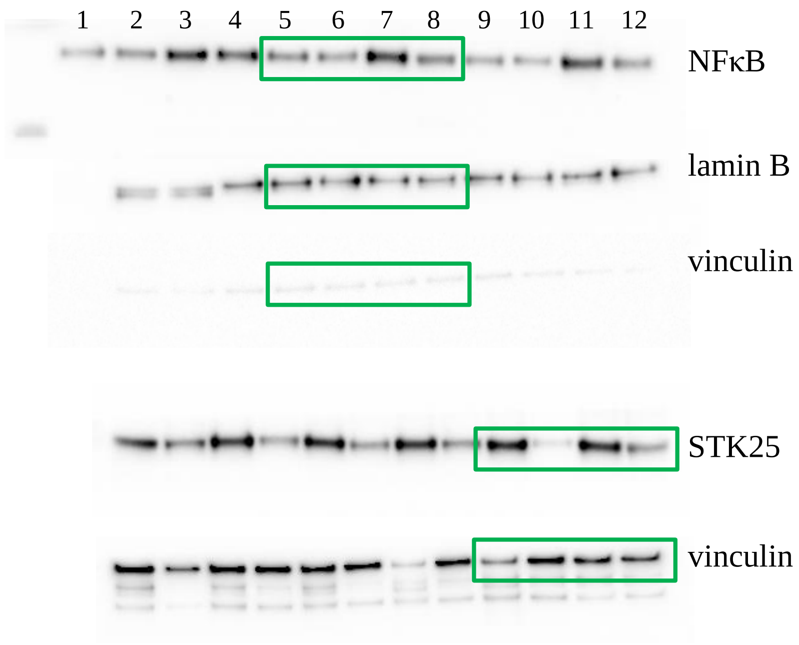

Green boxes indicates part
shown in each respective figure

Figure 2i

1. NTC siRNA basal
2. *STK25* siRNA basal
3. NTC siRNA treated
4. *STK25* siRNA treated
5. NTC siRNA basal
6. *STK25* siRNA basal
7. NTC siRNA treated
8. *STK25* siRNA treated
9. NTC siRNA basal
10. *STK25* siRNA basal
11. NTC siRNA treated
12. *STK25* siRNA treated

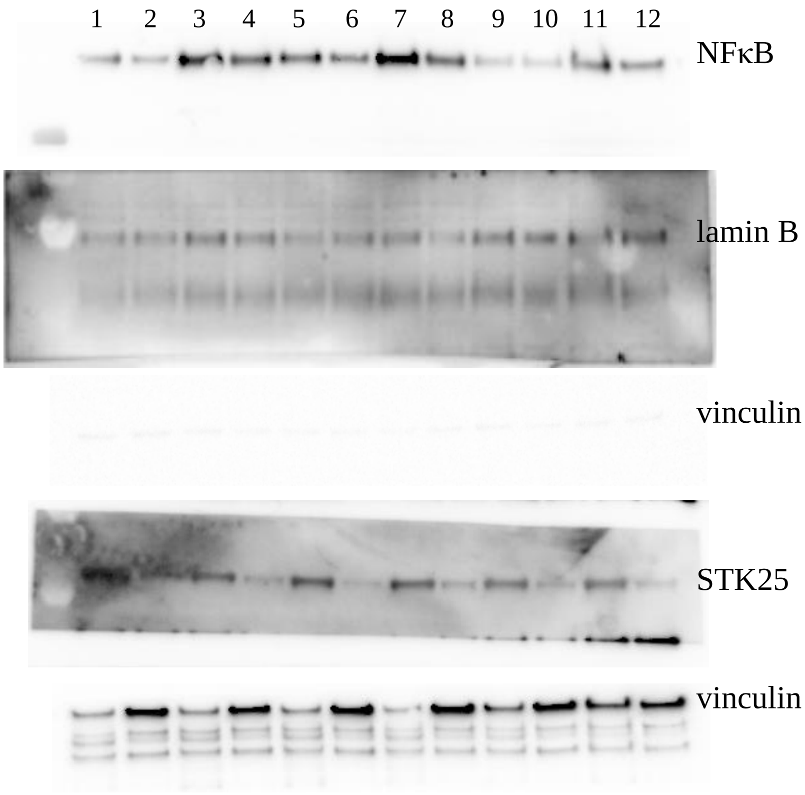

1. NTC siRNA basal
2. *STK25* siRNA basal
3. NTC siRNA treated
4. *STK25* siRNA treated
5. NTC siRNA basal
6. *STK25* siRNA basal
7. NTC siRNA treated
8. *STK25* siRNA treated
9. NTC siRNA basal
10. *STK25* siRNA basal
11. NTC siRNA treated
12. *STK25* siRNA treated

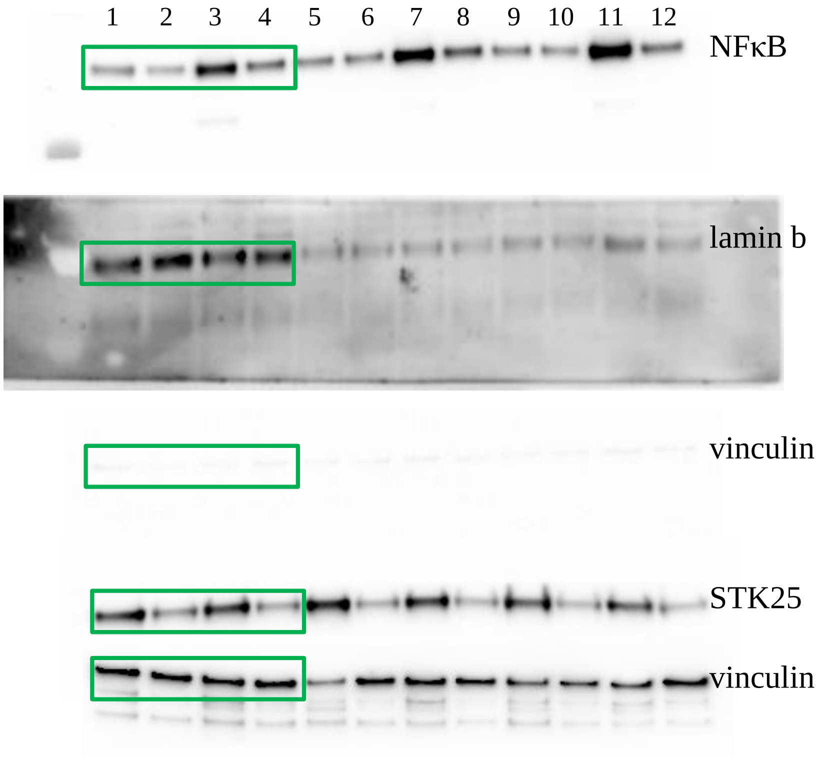

Green boxes indicates part shown in each respective figure

Figure 6f

1. NTC siRNA basal
2. *STK25* siRNA basal
3. NTC siRNA treated
4. *STK25* siRNA treated
5. NTC siRNA basal
6. *STK25* siRNA basal
7. NTC siRNA treated
8. *STK25* siRNA treated
9. NTC siRNA basal
10. *STK25* siRNA basal
11. NTC siRNA treated
12. *STK25* siRNA treated

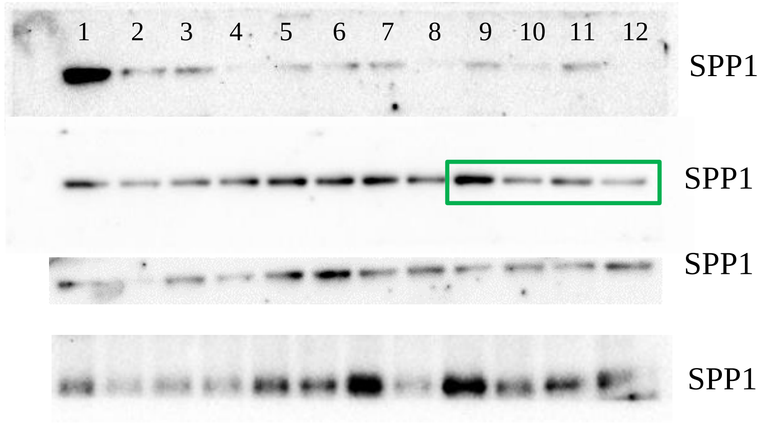

1. NTC siRNA basal
2. *STK25* siRNA basal
3. NTC siRNA treated
4. *STK25* siRNA treated
5. NTC siRNA basal
6. *STK25* siRNA basal
7. NTC siRNA treated
8. *STK25* siRNA treated
9. NTC siRNA basal
10. *STK25* siRNA basal
11. NTC siRNA treated
12. *STK25* siRNA treated

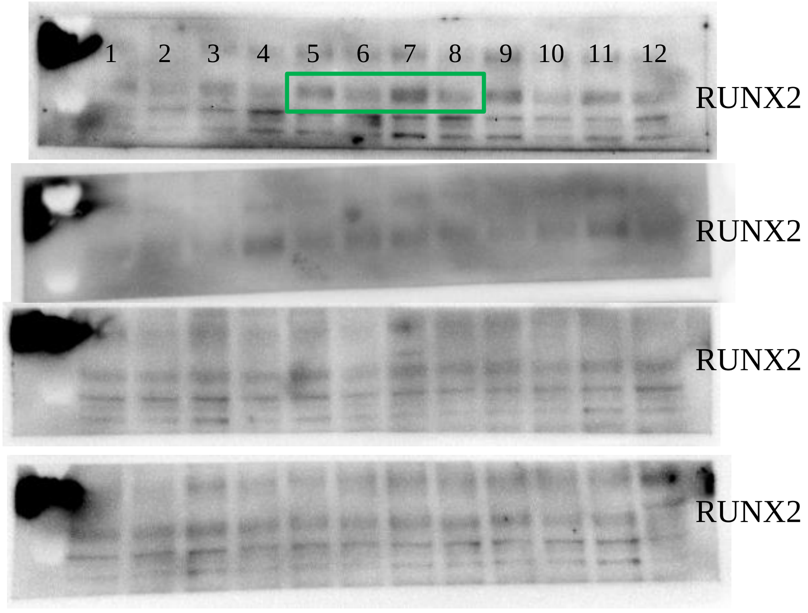

1. NTC siRNA basal
2. *STK25* siRNA basal
3. NTC siRNA treated
4. *STK25* siRNA treated
5. NTC siRNA basal
6. *STK25* siRNA basal
7. NTC siRNA treated
8. *STK25* siRNA treated
9. NTC siRNA basal
10. *STK25* siRNA basal
11. NTC siRNA treated
12. *STK25* siRNA treated

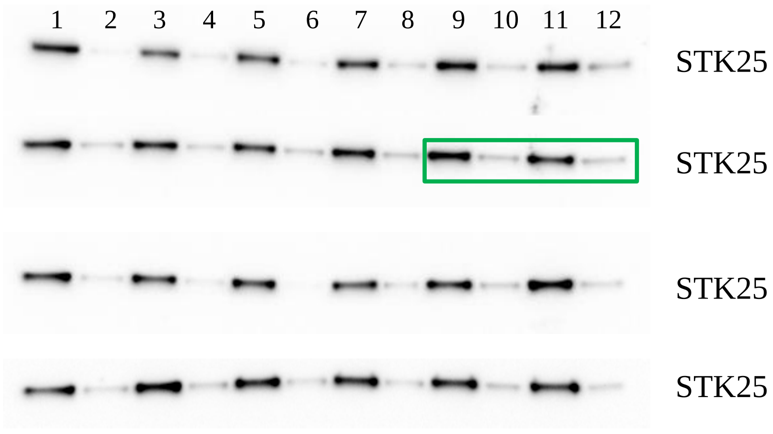

Green boxes indicates part shown in each respective figure

Figure 6f cont.

1. NTC siRNA basal
2. *STK25* siRNA basal
3. NTC siRNA treated
4. *STK25* siRNA treated
5. NTC siRNA basal
6. *STK25* siRNA basal
7. NTC siRNA treated
8. *STK25* siRNA treated
9. NTC siRNA basal
10. *STK25* siRNA basal
11. NTC siRNA treated
12. *STK25* siRNA treated

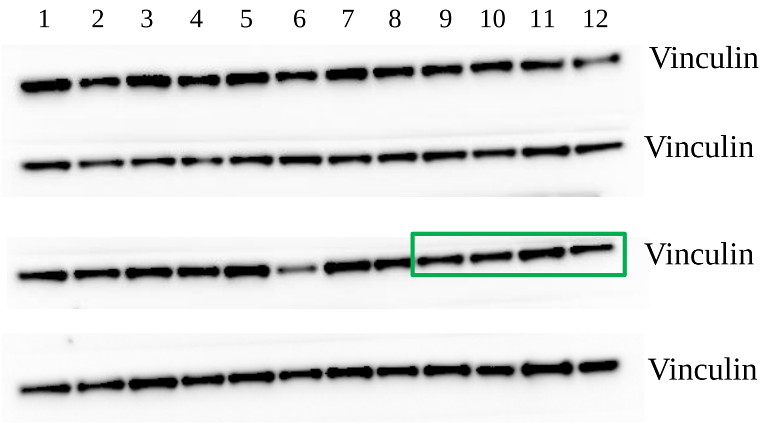

Green boxes indicates part shown in each respective figure

Supplementary Figure 3a

1. mASMC NTC siRNA basal
2. mASMC *Stk25* siRNA basal
3. mASMC NTC siRNA treated
4. mASMC *Stk25* siRNA treated
5. mASMC NTC siRNA basal
6. mASMC *Stk25* siRNA basal
7. mASMC NTC siRNA treated
8. mASMC *Stk25* siRNA treated
9. mASMC NTC siRNA basal
10. mASMC *Stk25* siRNA basal
11. mASMC NTC siRNA treated
12. mASMC *Stk25* siRNA treated

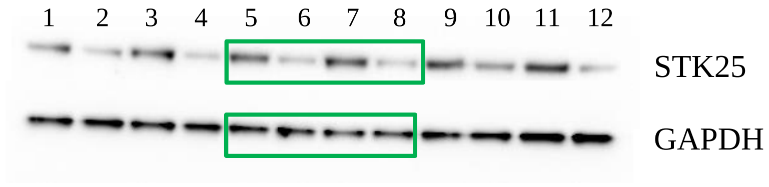

1. mASMC NTC siRNA basal
2. mASMC *Stk25* siRNA basal
3. mASMC NTC siRNA treated
4. mASMC *Stk25* siRNA treated
5. mAEC NTC siRNA basal
6. mAEC *Stk25* siRNA basal
7. mAEC NTC siRNA treated
8. mAEC *Stk25* siRNA treated
9. mAEC NTC siRNA basal
10. mAEC *Stk25* siRNA basal
11. mAEC NTC siRNA treated
12. mAEC *Stk25* siRNA treated

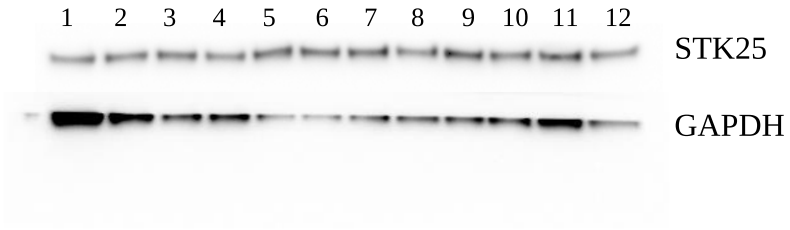

1. mAEC NTC siRNA basal
2. mAEC *Stk25* siRNA basal
3. mAEC NTC siRNA treated
4. mAEC *Stk25* siRNA treated
5. mAEC NTC siRNA basal
6. mAEC *Stk25* siRNA basal
7. mAEC NTC siRNA treated
8. mAEC *Stk25* siRNA treated
9. mAEC NTC siRNA basal
10. mAEC *Stk25* siRNA basal
11. mAEC NTC siRNA treated
12. mAEC *Stk25* siRNA treated

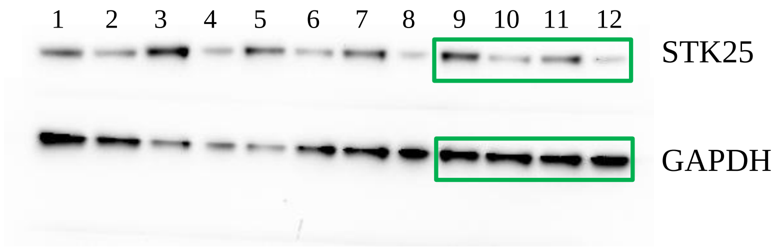

Green boxes indicates part shown in each respective figure

Supplementary Figure 5

1. NTC siRNA basal
2. *STK25* siRNA basal
3. NTC siRNA treated
4. *STK25* siRNA treated
5. NTC siRNA basal
6. *STK25* siRNA basal
7. NTC siRNA treated
8. *STK25* siRNA treated
9. NTC siRNA basal
10. *STK25* siRNA basal
11. NTC siRNA treated
12. *STK25* siRNA treated

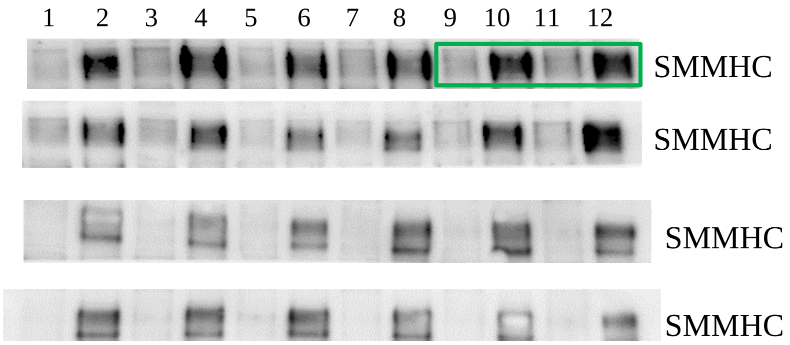

1. NTC siRNA basal
2. *STK25* siRNA basal
3. NTC siRNA treated
4. *STK25* siRNA treated
5. NTC siRNA basal
6. *STK25* siRNA basal
7. NTC siRNA treated
8. *STK25* siRNA treated
9. NTC siRNA basal
10. *STK25* siRNA basal
11. NTC siRNA treated
12. *STK25* siRNA treated

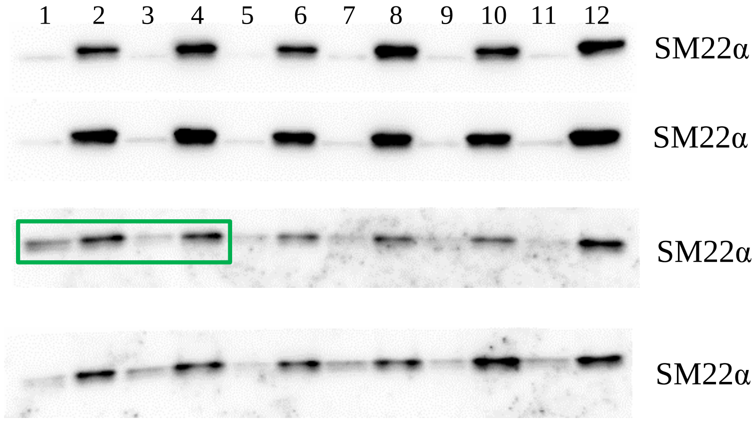

1. NTC siRNA basal
2. *STK25* siRNA basal
3. NTC siRNA treated
4. *STK25* siRNA treated
5. NTC siRNA basal
6. *STK25* siRNA basal
7. NTC siRNA treated
8. *STK25* siRNA treated
9. NTC siRNA basal
10. *STK25* siRNA basal
11. NTC siRNA treated
12. *STK25* siRNA treated

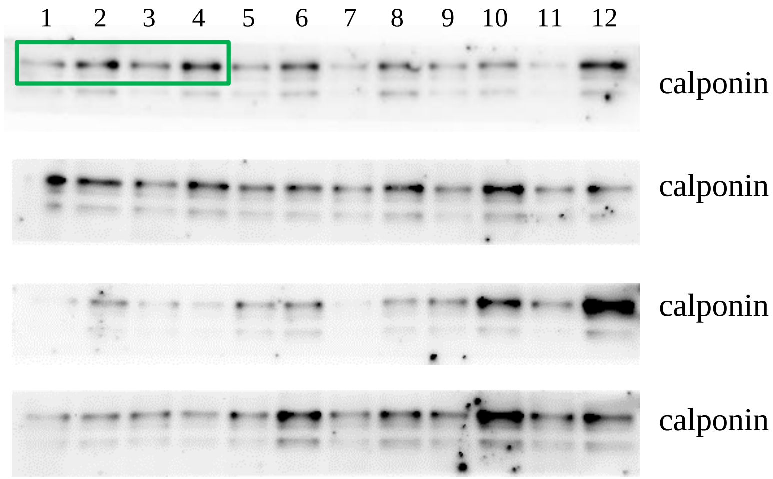

Green boxes indicates part shown in each respective figure

Supplementary Figure 5 cont.

1. NTC siRNA basal
2. *STK25* siRNA basal
3. NTC siRNA treated
4. *STK25* siRNA treated
5. NTC siRNA basal
6. *STK25* siRNA basal
7. NTC siRNA treated
8. *STK25* siRNA treated
9. NTC siRNA basal
10. *STK25* siRNA basal
11. NTC siRNA treated
12. *STK25* siRNA treated

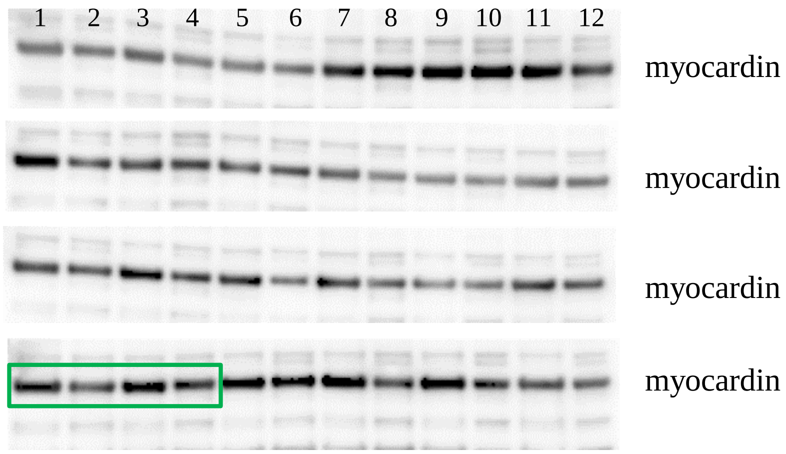

1. NTC siRNA basal
2. *STK25* siRNA basal
3. NTC siRNA treated
4. *STK25* siRNA treated
5. NTC siRNA basal
6. *STK25* siRNA basal
7. NTC siRNA treated
8. *STK25* siRNA treated
9. NTC siRNA basal
10. *STK25* siRNA basal
11. NTC siRNA treated
12. *STK25* siRNA treated

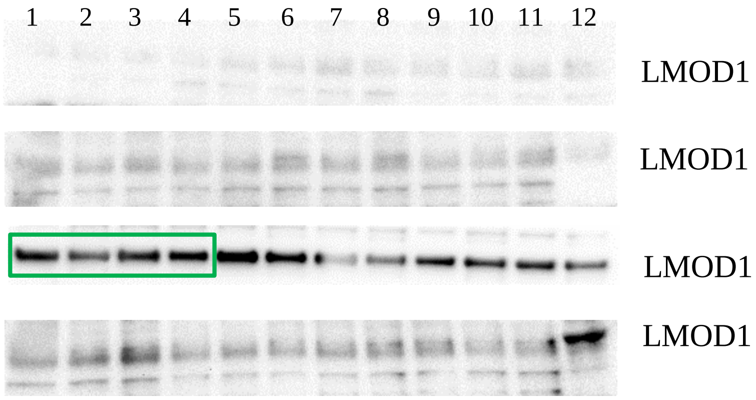

1. NTC siRNA basal
2. *STK25* siRNA basal
3. NTC siRNA treated
4. *STK25* siRNA treated
5. NTC siRNA basal
6. *STK25* siRNA basal
7. NTC siRNA treated
8. *STK25* siRNA treated
9. NTC siRNA basal
10. *STK25* siRNA basal
11. NTC siRNA treated
12. *STK25* siRNA treated

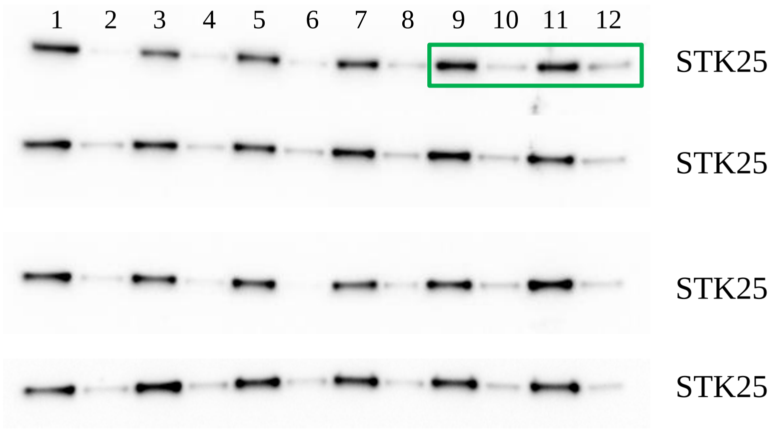

Green boxes indicates part shown in each respective figure

Supplementary Figure 5 cont.

1. NTC siRNA basal
2. *STK25* siRNA basal
3. NTC siRNA treated
4. *STK25* siRNA treated
5. NTC siRNA basal
6. *STK25* siRNA basal
7. NTC siRNA treated
8. *STK25* siRNA treated
9. NTC siRNA basal
10. *STK25* siRNA basal
11. NTC siRNA treated
12. *STK25* siRNA treated

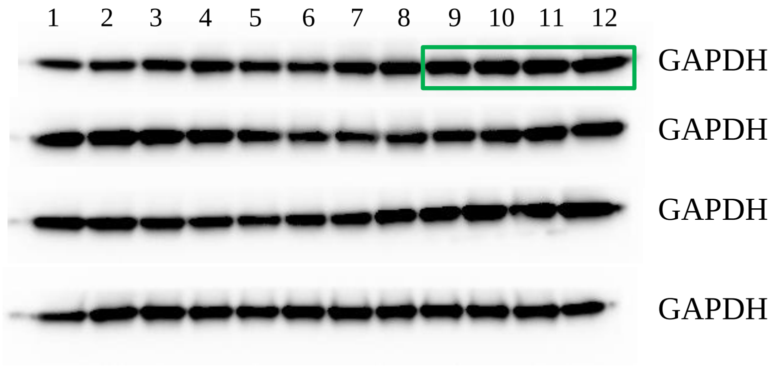

Green boxes indicates part
shown in each respective figure

Supplementary Figure 10a

1-2. NTC siRNA
3-4. *STK25* siRNA
5-6. NTC siRNA
7-8. *STK25* siRNA
9-10. NTC siRNA
11-12. *STK25* siRNA

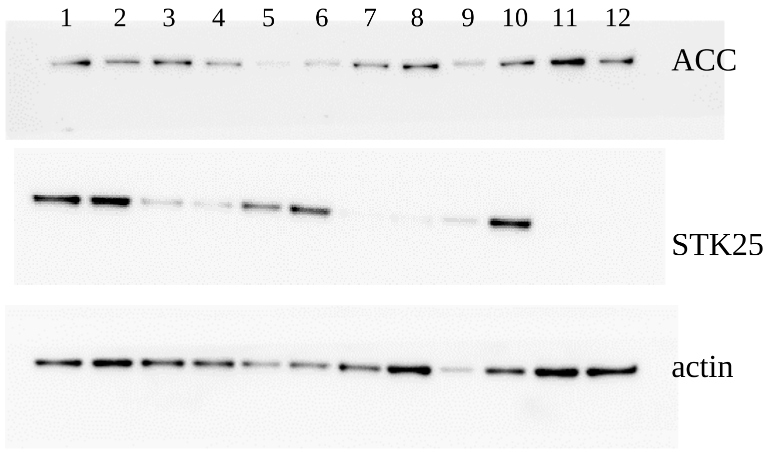

1. NTC siRNA
2. *STK25* siRNA
3. NTC siRNA
4. *STK25* siRNA
5. NTC siRNA
6. *STK25* siRNA

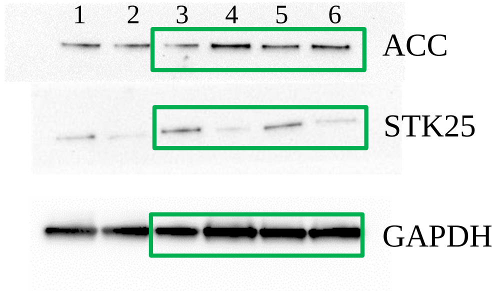

Supplementary Figure 10b

1. NTC siRNA
2. *STK25* siRNA
3. NTC siRNA
4. *STK25* siRNA
5. NTC siRNA
6. *STK25* siRNA
7. NTC siRNA
8. *STK25* siRNA
9. NTC siRNA
10. *STK25* siRNA
11. NTC siRNA
12. *STK25* siRNA

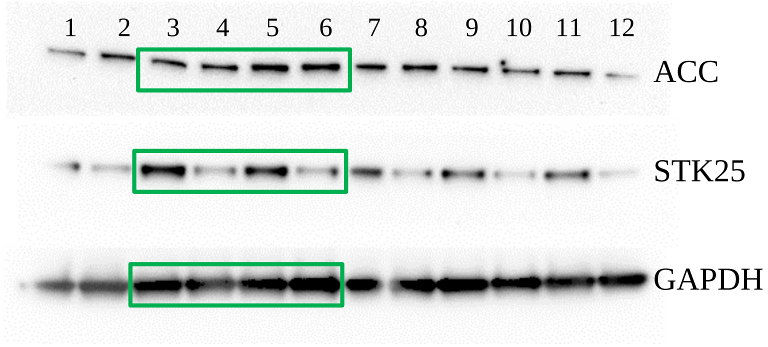

Green boxes indicates part
shown in each respective figure

Supplementary Table 1. List of antibodies used for Western blot and immunofluorescence analysis

Type	Antibody name and catalogue number	Working dilution	Company
Primary antibodies	anti-STK25 (25821-1-AP)	1:750	Proteintech (Chicago, IL)
	anti-GAPDH (sc-47724)	1:1000	Santa Cruz Biotechnology (Santa Cruz, CA)
	anti-NFκB (ab16502)	1:500	Abcam (Cambridge, UK)
	anti-lamin B (ab229025)	1:1000	Abcam
	anti-vinculin (sc-25336)	1:1000	Santa Cruz Biotechnology
	anti-8-oxoG (ab62623)	1:200	Abcam
	anti-E06 (J0914)	1:100	Avanti Polar Lipids, Inc. (Alabaster, AL)
	anti-KDEL (ab176333)	1:1000	Abcam
	anti-CHOP (MA1-250)	1:200	Invitrogen (Waltman, MA)
	anti-Ki67 (14-5698-82)	1:200	Invitrogen
	anti-SMMHC (ab125884)	1:1000	Abcam
	anti-SM22α (PA5-29767)	1:1000	Invitrogen
	anti-calponin (MA5-11620)	1:1000	Invitrogen
	anti-myocardin (SAB4200539)	1:1000	Sigma-Aldrich (St. Louis, MO)
	anti-LMOD1 (HPA028325)	1:1000	Sigma Aldrich
	anti-SPP1 (PA5-34579)	1:1000	Invitrogen
	anti-RUNX2 (ab23981)	1:500	Abcam
	anti-ACC (#3662)	1:1000	Cell Signaling Technology (Boston, MA)
	Mouse IgG2b Isotype Control (14-4732-82)	1:100	Invitrogen
	Mouse IgM Isotype Control (14-4752-81)	1:67	Invitrogen
	Rabbit IgG Isotype Control (31235)	1:24000	Invitrogen
Secondary antibodies	Donkey anti-mouse IgG, Alexa Fluor 488 (A21202)	1:500	Invitrogen
	Goat anti-rabbit IgG, Alexa Fluor 488 (A11008)	1:500	Invitrogen
	Donkey anti-rabbit IgG, Alexa Fluor 594 (A21207)	1:500	Invitrogen
	Goat anti-mouse IgG, Alexa Fluor 594 (A11005)	1:500	Invitrogen
	Goat anti-rat IgG, Alexa Fluor 594 (A11007)	1:500	Invitrogen
	Goat anti-rabbit IgG, HRP-linked (#7074)	1:1000	Cell Signaling Technology
	Horse anti-mouse IgG, HRP-linked (#7076)	1:1000	Cell Signaling Technology

Supplementary Table 2. Differential regulation of the total protein abundance in hASMCs transfected with NTC or *STK25* siRNA and treated with oleic acid and oxLDL for 24 hours

Accession number	Symbol	Name	*P* value	Ratio	Function
P02788	LTF	Lactotransferrin	0.004	-1.87	Lactoferrin (EC 3.4.21) is an iron-binding glycoprotein of the transferrin family that is expressed in most biologic fluids and is a major component of mammals' innate immune system. It has also been shown to increase bone formation.
P02771	AFP	Alpha-fetoprotein	0.001	-1.72	The AFP gene is a member of a multigenic family that comprises the related genes encoding albumin, afamin, and vitamin D-binding protein. It is involved in proliferation and is increased in several types of cancers.
P02768	ALB	Albumin	0.002	-1.71	Albumin is the major protein of the blood plasma, supporting the oncotic pressure. It is also an important circulating antioxidant and possesses enzymatic properties.
P26022	PTX3	Pentraxin-related protein PTX3	1.2E-5	-1.67	PTX3, the prototypic long pentraxin, is a soluble pattern recognition receptor activated by cytokines, that is involved in the regulation of innate resistance to pathogens and inflammatory reactions. It has been suggested as a biomarker for inflammatory vascular disease.
P02765	AHSG	Alpha-2-HS-glycoprotein	0.005	-1.65	The AHSG gene encodes a multifunctional phosphorylated extracellular glycoprotein that has an important role in inhibition of vascular calcium deposition. It also promotes endocytosis, possesses opsonic properties and influences the mineral phase of bone.
P02774	DBP	Vitamin D-binding protein	0.002	-1.62	DPB (EC 6.3.1.5) is a transcriptional activator recognizes and binds to promoters of genes such as albumin. The major function of DBP is binding, solubilization, and transport of vitamin D and its metabolites.
P05543	SERPINA7	Thyroxine-binding globulin	0.002	-1.60	The SERPINA7 gene is a member of the serpin gene family. SERPINA7 [thyroxine-binding globulin (TBG)] is the major thyroid hormone transport protein in serum.
Q9BZI7	UPF3B	Regulator of nonsense transcripts 3B	0.002	-1.60	UPF3B is a component of an exon-junction complex that promotes nonsense-mediated decay, which is a mechanism for degrading transcripts with premature termination codons arising from errors in transcription or splicing.
P61599	NAA20	N-alpha-acetyltransferase 20	0.003	-1.57	NAA20 (EC 2.3.1.254) is a component of N-acetyltransferase complex B, involved in regulation of cell proliferation and cellular migration.
Q9NQS7	INCENP	Inner centromere protein	0.004	-1.54	INCENP is a component of the chromosome passenger complex, a complex that acts as a key regulator of mitosis, and is also involved in regulating cell proliferation.
P19823	ITIH2	Inter-alpha-trypsin inhibitor heavy chain H2	2.0E-5	-1.54	The inter-alpha-trypsin inhibitors are a family of structurally related plasma serine protease inhibitors involved in extracellular matrix stabilization and in prevention of tumor metastasis.
Q15283	RASA2	Ras GTPase-activating protein 2	0.0007	1.52	Ras signaling is involved in several cellular processes. RASA2 hydrolyze GTP to GDP on Ras and thereby inactivating it, which leads to decreased proliferation and differentiation.
P35443	THBS4	Thrombospondin-4	0.04	-1.52	The thrombospondins are a family of extracellular calcium binding proteins involved in cell proliferation, adhesion, and migration.
P49747	COMP	Cartilage oligomeric matrix protein	0.002	-1.51	COMP is a pentameric extracellular matrix protein that catalyzes the assembly of collagens and promotes formation of well-defined fibrils.
Q9NSY0	NRBP2	Nuclear receptor-binding protein 2	0.005	-1.50	NRBP2 is considered an autophagy-related gene, and may regulate apoptosis of neural progenitor cells during their differentiation. It has been shown to regulate cell proliferation and migration.

Accession number	Symbol	Name	*P* value	Ratio	Gene function
P05121	SERPINE1	Plasminogen activator inhibitor 1	8.7E-7	-1.49	The SERPINE1 gene is a member of the serpin gene family. SERPINE1 (PAI1) inhibits the conversion of plasminogen into plasmin, which breaks down fibrin clots. Thus, SERPINE1 negatively regulates fibrinolysis and impairs the dissolution of clots. It has also been shown to regulate migration and proliferation.
P23677	ITPKA	Inositol-trisphosphate 3-kinase A	0.03	-1.49	ITPKA (EC 2.7.1.127) catalyzes the phosphorylation of Ins(1,4,5)P3 to Ins(1,3,4,5)P4, both of which are modulators of calcium homeostasis. ITPKA has been linked to migration via its binding to actin.
P01009	SERPINA1	Alpha-1-antitrypsin	0.003	-1.47	The SERPINA1 gene is a member of the serpin gene family. The SERPINA1 gene encodes alpha-1-antitrypsin. It is known to decrease coagulation time and has proteolytic activity against insulin and plasmin, but it has also been shown to regulate migration.
Q96QB1	DLC1	Rho GTPase-activating protein 7	1.6E-6	1.46	RhoGAPs are GTPase activating proteins. DLC1 is a RhoGAP that is regulating cell proliferation, adhesion, and migration. It's known to be a tumor suppressor.
P20742	PZP	Pregnancy zone protein	0.004	-1.44	PZP is a protease inhibitor and one of the major pregnancy-associated plasma proteins. PZP is believed to play a role in immune-regulation, but the specific biological significance of PZP is yet to be determined.
Q96IF1	AJUBA	LIM domain-containing protein ajuba	0.0002	1.44	AJUBA contribute to cell fate determination via regulation of the Hippo pathway and control cell proliferation and differentiation.
Q15048	LRRC14	Leucine-rich repeat-containing protein 14	0.003	-1.43	Members of the LRRC superfamily are diverse in respect to their structure and are involved via protein–protein interactions in a variety of functions including DNA repair, chromosomal stability and heart development. It is known to negatively regulate Toll-like receptor-mediated NFκB signaling.
P00450	CP	Ceruloplasmin	0.01	-1.43	Ceruloplasmin (EC 1.16.3.1) is a plasma metalloprotein involved in iron homeostasis. It has been shown to enhances LDL oxidation and decreases nitric oxide NO bioavailability through nitric oxidase activity.
P36955	SERPINF1	Pigment epithelium-derived factor	0.0004	-1.42	The SERPINF1 gene is a member of the serpin gene family and encodes PEDF. PEDF has a variety of functions including proliferation and inhibition of angiogenesis.
Q2NKX8	ERCC6L	DNA excision repair protein ERCC-6-like	0.004	-1.42	ERCC6L (EC 3.6.4.12) is a DNA helicase and an essential component of the spindle assembly checkpoint. It has been shown to stimulate cancer cell proliferation.
Q9H2F3	HSD3B7	3 beta-hydroxysteroid dehydrogenase type 7	1.4E-5	1.41	The 3-beta-HSD enzymatic system plays a crucial role in the biosynthesis of all classes of hormonal steroids. The HSD3B7 enzyme (EC 1.1.1.181) catalyzes two reactions required for the inversion of the 3-hydroxyl group of cholesterol to the 3-hydroxyl of bile acids.
P04278	SHBG	Sex hormone-binding globulin	0.01	-1.41	SHBG functions as an androgen transport protein, but may also be involved in receptor mediated processes. It is specific for 5-alpha-dihydrotestosterone, testosterone, and 17-beta-estradiol, and regulates the plasma metabolic clearance rate of steroid hormones by controlling their plasma concentration.
O00592	PODXL	Podocalyxin	0.002	1.41	PODXL is an adhesion protein involved in migration.
O96028	NSD2	Histone-lysine N-methyltransferase NSD2	0.03	-1.41	NSD2 (EC 2.1.1.43) is a histone lysine methyltransferase functioning as a transcriptional regulator. By adding a methyl group to histones, histone methyltransferases can turn off the activity of certain genes.

A ratio of 1.4-fold serves as the threshold for differential regulation. The functions of the differentially expressed proteins were annotated according to Gene Ontology database,[1] NCBI OMIM and/or PubMed

Supplementary Table 3. Differential regulation of the phosphorylation pattern in hASMCs transfected with NTC or *STK25* siRNA and treated with oleic acid and oxLDL for 24 hours

Accession number	Symbol	Name	Phosphory-lation site	*P* value	Ratio	Function
P02452	COL1A1	Collagen alpha-1 chain	S1023*	5.6E-08	1.72	Collagen is a protein that strengthens and supports many tissues in the body, including cartilage, bone, tendon, and skin. The COL1A1 gene produces a component of type I collagen.
Q9H6R7	WDCP	WD repeat and coiled-coil-containing protein	S690*	0.005	-1.65	The function of WDCP is still elusive, but it is known to be a fusion partner of anaplastic lymphoma kinase which is involved in chromosomal rearrangement and cancer development.
Q9Y6I9.2	TEX264	Testis-expressed protein 264	S269*<br>S272*	0.03	-1.65	TEX264 is an ER-phagy receptor involved in ERAD and ER stress.
O75477	ERLIN1	Erlin-1	S336*	0.0001	-1.55	Component of the ERLIN1/ERLIN2 complex which mediates ERAD of inositol 1,4,5-trisphosphate receptors. It is also involved in regulation of cellular cholesterol homeostasis by regulation the SREBP signaling pathway.
P02452.6	COL1A1	Collagen alpha-1 chain	S363	0.0005	1.55	Collagen is a protein that strengthens and supports many tissues in the body, including cartilage, bone, tendon, and skin. The COL1A1 gene produces a component of type I collagen.
Q9NZ72	STMN3	Stathmin-3	S50*	0.0004	1.54	STMN3 is involved in cytoskeleton dynamics where it exhibits microtubule-destabilizing activity.
Q96EV8.2	DTNBP1	Dysbindin	S316*<br>S321*	0.02	-1.53	Dysbindin is a component of the BLOC-1 complex, a complex that is required for normal biogenesis of lysosome-related organelles, such as platelet dense granules and melanosomes. In concert with the AP-3 complex, the BLOC-1 complex is required to target membrane protein cargos into vesicles assembled at cell bodies for delivery.
Q8TCJ2	STT3B	Dolichyl-diphosphooligosaccharide-protein glycosyltransferase subunit STT3B	S498*	0.003	1.53	STT3B (EC 2.4.99.18) plays a role in the ERAD pathway that mediates ubiquitin-dependent degradation of misfolded endoplasmic reticulum proteins by mediating N-glycosylation of unfolded proteins, which are then recognized by the ERAD pathway and targeted for degradation.
O94888.1	UBXN7	UBX domain-containing protein 7	S280*	0.005	-1.52	UBXN7 is a cofactor protein that provides a scaffold for different ubiquitin ligase complexes. It is known to decrees the levels of NRF2, an important transcription factor involved in protection against oxidative stress.
Q8IWB9	TEX2	Testis-expressed protein 2	T742*	0.04	-1.48	Not much is known about TEX2, but it suggested to be a ceramide transporter.
Q13111.2	CHAF1A	Chromatin assembly factor 1 subunit A	Position unknown	0.001	-1.48	A core component of the CAF-1 complex, a complex mediating chromatin assembly.
Q16658	FSCN1	Fascin	S127*	0.0003	1.48	Fascin is an actin-binding protein. It organizes filamentous actin into parallel bundles and plays a role in cell motility and migration.
P02452.4	COL1A1	Collagen alpha-1 chain	S546*	0.0003	1.48	Collagen is a protein that strengthens and supports many tissues in the body, including cartilage, bone, tendon, and skin. The COL1A1 gene produces a component of type I collagen.

Accession number	Symbol	Name	S558	*P* value	Ratio	Function
Q12797.1	ASPH	Aspartyl/asparaginyl beta-hydroxylase	S558	0.004	1.47	ASPH (EC 1.14.11.16) belongs to the aspartyl/asparaginyl beta-hydroxylase family and specifically hydroxylates an Asp or Asn residue. It regulates epidermal growth factor-like domains of a number of proteins and controls calcium homeostasis.
P23497.3	SP100	Nuclear autoantigen Sp-100	T185	0.02	-1.46	SP100 is a transcriptional and chromatin regulatory factor mainly involved in inhibition of viral transcription.
Q8IV32	CCDC71	Coiled-coil domain-containing protein 71	S208*	0.02	-1.45	The function of CCDC71 is still unknown.
Q8IYB3.21	SRRM1	Serine/arginine repetitive matrix protein 1	S494*	0.003	-1.45	SRRM1 regulates alternative splicing and have been shown to be involved in proliferation and migration.
Q53EP0.4	FNDC3B	Fibronectin type III domain-containing protein 3B	S981	1.8E-06	1.45	FNDC3B regulates the differentiation of adipocytes and osteoblasts and has been shown to regulate the proliferation and metastatic capacity of tumor cells.
Q13620; Q13619	CUL4B	Cullin-4B	S468	9.9E-06	1.44	The CUL4B gene encodes a scaffold protein of the cullin 4B-RING ubiquitin ligase (E3) complex that regulates degradation of cellular proteins. It has been shown to be involved in proliferation.
Q8WUH2	TGFBRAP1	Transforming growth factor-beta receptor-associated protein 1	S854*	0.02	-1.44	This gene encodes a protein that binds to TGF-β receptors and plays a role in TGF-β signaling, which is involved in many cellular processes. The encoded protein acts as a chaperone in signaling downstream of TGF-β.
Q96EY5.1	MVB12A	Multivesicular body subunit 12A	S170*	0.03	-1.43	Component of the ESCRT-I complex, a regulator required for the sorting of endocytic ubiquitinated cargos into multivesicular bodies, and thereby controlling several cellular processes.
O60341.3	KDM1A	Lysine-specific histone demethylase 1A	S137*	0.048	-1.43	KDM1A (EC 1.14.99.66) is a histone demethylase. Histone demethylases are a family of enzymes that catalyze the removal of methyl groups from lysine and arginine residues on histone tails. It is involved in regulation of several cellular processes.
Q9Y2K7.2	KDM2A	Lysine-specific demethylase 2A	Position unknown	0.048	-1.43	KDM2A (EC 1.14.11.27) is a histone demethylase. It is involved in regulation of several cellular processes. May also recognize and bind to some phosphorylated proteins and promote their ubiquitination and degradation.
Q9NYU1; Q9NYU2	UGGT2	UDP-glucose:glycoprotein glucosyltransferase 2	Position unknown	0.0004	1.43	UGGT2 (EC 2.4.1) recognizes glycoproteins with minor folding defects and recycles them back to the endoplasmic reticulum for refolding or degradation, thus providing quality control for protein folding in the ER.
Q9Y2U8.3	LEMD3	Inner nuclear membrane protein Man1	S556	1.5E-05	1.43	LEMD3 is an integral protein of the inner nuclear membrane. It functions as a specific repressor of TGF-β and BMP signaling through its interaction with the SMAD proteins.
P24386	CHM	Rab proteins geranylgeranyltransferase component A 1	Position unknown	0.01	1.43	The CHM gene encodes the component A of the Rab escort protein-1 which encodes a chaperone protein for the prenylation of Rab GTPases, which regulates intracellular vesicular trafficking, and thereby controls cellular processes such as adhesion, migration, and proliferation.

Accession number	Symbol	Name	S196	*P* value	Ratio	Function
Q8N3E9	PLCD3	1-phosphatidylinositol 4,5-bisphosphate phosphodiesterase delta-3	S196	0.0001	1.42	PLCD3 (EC 3.1.4.11) hydrolyzes the phosphatidylinositol 4,5-bisphosphate to generate the two second messenger molecules diacylglycerol and inositol 1,4,5-trisphosphate, which mediate a wide variety of cellular stimuli.
Q6ZRS2.5	SRCAP	Helicase SRCAP	S1859*	0.007	-1.42	SRCAP (EC 3.6.1) is a catalytic component of a complex which mediates the ATP-dependent exchange of histone H2AZ/H2B dimers for nucleosomal H2A/H2B, leading to transcriptional regulation of selected genes by chromatin remodeling.
P45974.1	USP5	Ubiquitin carboxyl-terminal hydrolase 5	S785*	0.004	-1.42	USP5 (EC 3.4.19.12) is a protease involved in unanchored Lys-48-linked polyubiquitin disassembly. Cleaves linear and branched multiubiquitin polymers.
Q71F23	CENPU	Centromere protein U	T98*	0.006	-1.41	CENPU is a component of the CENPA-NAC complex, a complex that plays a central role in assembly of kinetochore proteins, mitotic progression and chromosome segregation.
Q96P11; Q63ZY6	NSUN5	28S rRNA (cytosine-C(5))-methyltransferase	S327*	0.002	-1.41	NSUN5 (EC 2.1.1) is a methyltransferase involved in several different cellular processes.
P41970	ELK3	ETS domain-containing protein Elk-3	S396*	8.3E-05	-1.41	ELK3 is a transcription factor regulating the expression of many genes. Under basal conditions, ELK3 is a transcriptional repressor, which converts to a transcriptional activator in response to RAS/ERK signaling and is involved in cell migration, angiogenesis, and malignant progression.
Q96C24.1	SYTL4	Synaptotagmin-like protein 4	S201*	0.049	-1.41	SYTL4, also known as granuphilin, is involved in intracellular membrane trafficking where it is known to assist in the docking of granules to the plasma membrane.
Q9H3G5	CPVL	Probable serine carboxypeptidase CPVL	S32	8.0E-06	1.41	CPVL (EC 3.4.16) may be involved in the digestion of phagocytosed particles in the lysosome, participation in an inflammatory protease cascade, and trimming of peptides for antigen presentation.

A ratio of 1.4-fold serves as the threshold for differential regulation. The functions of the differentially phosphorylated proteins were annotated according to Gene Ontology database,[1] NCBI OMIM and/or PubMed

*Phosphosites annotated in PhosphoSitePlus[2]

REFERENCES

1 Ashburner, M. et al. Gene ontology: tool for the unification of biology. The Gene Ontology Consortium. Nat Genet 25, 25-29, doi:10.1038/75556 (2000).

2 Hornbeck, P. V. et al. PhosphoSitePlus, 2014: mutations, PTMs and recalibrations. Nucleic Acids Res 43, D512-D520, doi:10.1093/nar/gku1267 (2015).
