# Supplementary material for: Silencing of STE20-type kinase STK25 in human aortic endothelial and smooth muscle cells is atheroprotective
Source: Commun Biol. 2022 Apr 19;5:379. doi: 10.1038/s42003-022-03309-9 (PMC9018782; doi:10.1038/s42003-022-03309-9)
Supplement: Supplementary file 5 — Reporting summary [file 42003_2022_3309_MOESM5_ESM.pdf]

nature portfolio

Corresponding author(s): Margit Mahlapuu

Last updated by author(s): Feb 15, 2022

Reporting Summary

Nature Portfolio wishes to improve the reproducibility of the work that we publish. This form provides structure for consistency and transparency in reporting. For further information on Nature Portfolio policies, see our Editorial Policies and the Editorial Policy Checklist.

Statistics

For all statistical analyses, confirm that the following items are present in the figure legend, table legend, main text, or Methods section.

n/a	Confirmed	
☐	☒	The exact sample size (*n*) for each experimental group/condition, given as a discrete number and unit of measurement
☒	☐	A statement on whether measurements were taken from distinct samples or whether the same sample was measured repeatedly
☐	☒	The statistical test(s) used AND whether they are one- or two-sided<br>*Only common tests should be described solely by name; describe more complex techniques in the Methods section.*
☒	☐	A description of all covariates tested
☐	☒	A description of any assumptions or corrections, such as tests of normality and adjustment for multiple comparisons
☐	☒	A full description of the statistical parameters including central tendency (e.g. means) or other basic estimates (e.g. regression coefficient) AND variation (e.g. standard deviation) or associated estimates of uncertainty (e.g. confidence intervals)
☒	☐	For null hypothesis testing, the test statistic (e.g. *F*, *t*, *r*) with confidence intervals, effect sizes, degrees of freedom and *P* value noted<br>*Give P values as exact values whenever suitable.*
☒	☐	For Bayesian analysis, information on the choice of priors and Markov chain Monte Carlo settings
☒	☐	For hierarchical and complex designs, identification of the appropriate level for tests and full reporting of outcomes
☒	☐	Estimates of effect sizes (e.g. Cohen's *d*, Pearson's *r*), indicating how they were calculated

Our web collection on statistics for biologists contains articles on many of the points above.

Software and code

Policy information about availability of computer code

Data collection	The code and data for the enrichment analysis are available at https://bitbucket.org/scilifelab-lts/m_mahlapuu_2005/
Data analysis	The code and data for the enrichment analysis are available at https://bitbucket.org/scilifelab-lts/m_mahlapuu_2005/

For manuscripts utilizing custom algorithms or software that are central to the research but not yet described in published literature, software must be made available to editors and reviewers. We strongly encourage code deposition in a community repository (e.g. GitHub). See the Nature Portfolio guidelines for submitting code & software for further information.

Data

Policy information about availability of data

All manuscripts must include a data availability statement. This statement should provide the following information, where applicable:

- Accession codes, unique identifiers, or web links for publicly available datasets
- A description of any restrictions on data availability
- For clinical datasets or third party data, please ensure that the statement adheres to our policy

The phosphoproteomic datasets produced in this study are available in the PRIDE database (dataset identifier PXD031763). The source data for the graphs in the main figures are available in Supplementary Data 1.

Field-specific reporting

Please select the one below that is the best fit for your research. If you are not sure, read the appropriate sections before making your selection.

☒ Life sciences ☐ Behavioural & social sciences ☐ Ecological, evolutionary & environmental sciences

For a reference copy of the document with all sections, see nature.com/documents/nr-reporting-summary-flat.pdf

Life sciences study design

All studies must disclose on these points even when the disclosure is negative.

Sample size	Samples sizes were predetermined based on statistical power calculations or convention in the field. The exact sample size is given in the legend of each figure.
Data exclusions	Some qRT PCR data and cytokine/chemokine concentrations were excluded due to bad double values. Some Western blot bands were excluded due to bad running/hybridization.
Replication	All attempts at replication were successful. Findings in the human cells were also successfully reproduced in murine cells.
Randomization	For the cells, some wells are transfected with control siRNA and some with the target siRNA, but no further randomization possible. The mice are allocated to their respective group based on genotyping.
Blinding	The investigators were not blinded to treatment allocation. However, all analyses were fully standardized across all the samples to minimize any bias.

Reporting for specific materials, systems and methods

We require information from authors about some types of materials, experimental systems and methods used in many studies. Here, indicate whether each material, system or method listed is relevant to your study. If you are not sure if a list item applies to your research, read the appropriate section before selecting a response.

Materials & experimental systems

n/a	Involved in the study	
☐	☒	Antibodies
☐	☒	Eukaryotic cell lines
☒	☐	Palaeontology and archaeology
☐	☒	Animals and other organisms
☒	☐	Human research participants
☒	☐	Clinical data
☒	☐	Dual use research of concern

Methods

n/a	Involved in the study	
☒	☐	ChIP-seq
☒	☐	Flow cytometry
☒	☐	MRI-based neuroimaging

Antibodies

Antibodies used	All antibody information is listed in Supplementary Table 1.
Validation	The STK25 antibody has been validated by the company, as well as by us using knockout samples. All other antibodies have been validated by the respective company.

Eukaryotic cell lines

Policy information about cell lines

Cell line source(s)	Primary human aortic smooth muscle cells (hASMCs; SC6110; 3H Biomedical, Uppsala, Sweden). Primary human aortic endothelial cells (hAECs; 304-05A; Cell Applications Inc., Sigma-Aldrich, St. Louis, MO). Primary mouse aortic smooth muscle cells (mASMCs; C57-6080; Cell Biologics, Chicago, IL). Primary mouse aortic endothelial cells (mAECs; C57-6052; Cell Biologics). THP-1 monocytes (TIB-202; American Type Culture Collection, Manassas, VA).
Authentication	The cells were authenticated by the respective company.
Mycoplasma contamination	Cells were demonstrated to be free of mycoplasma infection by the MycoAlert Mycoplasma Detection Kit (LT07-218; Lonza, Basel, Switzerland).

Commonly misidentified lines<br>(See ICLAC register)	*Name any commonly misidentified cell lines used in the study and provide a rationale for their use.*

Animals and other organisms

Policy information about studies involving animals; ARRIVE guidelines recommended for reporting animal research

Laboratory animals	Mus musculus, C57BL/6, male, terminated at the age of 20 weeks.
Wild animals	N/A
Field-collected samples	N/A
Ethics oversight	All experiments were performed after prior approval from the local ethics committee.

Note that full information on the approval of the study protocol must also be provided in the manuscript.
